# Supplementary material for: Effects of Dietary Bacillus amyloliquefaciens SCAU-070 (Based on a Woody Plant-Based Diet) on Antioxidation, Immune and Intestinal Microbiota of Tilapia (Oreochromis niloticus)
Source: Microorganisms. 2024 May 23;12(6):1049. doi: 10.3390/microorganisms12061049 (PMC11205437; doi:10.3390/microorganisms12061049)
Supplement: Supplementary file 1 [file microorganisms-12-01049-s001.zip › microorganisms-2925219-supplementary.pdf]

Effects of Dietary *Bacillus amyloliquefaciens* SCAU-070 (Based on a Woody Plant-Based Diet) on Antioxidation, Immune and Intestinal Microbiota of Tilapia (*Oreochromis niloticus*)

Qijing Chen †, Feng Wu †, Xinye Chen, Qiaoting Yang, Biyin Ye, Xiaoyu Chen, Xiaoyong Zhang * and Qin Pan *

University Joint Laboratory of Guangdong Province, Hong Kong and Macao Region on Marine Bioresource Conservation and Exploitation, College of Marine Sciences, South China Agricultural University, Guangzhou 510642, China; chenqijing77@163.com (Q.C.); wufeng4505@163.com (F.W.); xinyeeast@stu.scau.edu.cn (X.C.); 13480325293@163.com (Q.Y.); y2564398@163.com (B.Y.); 13640009806@163.com (X.C.)

* Correspondence: zhangxiaoyong@scau.edu.cn (X.Z.); qpan@scau.edu.cn (Q.P.)

† These authors contributed equally to this work.

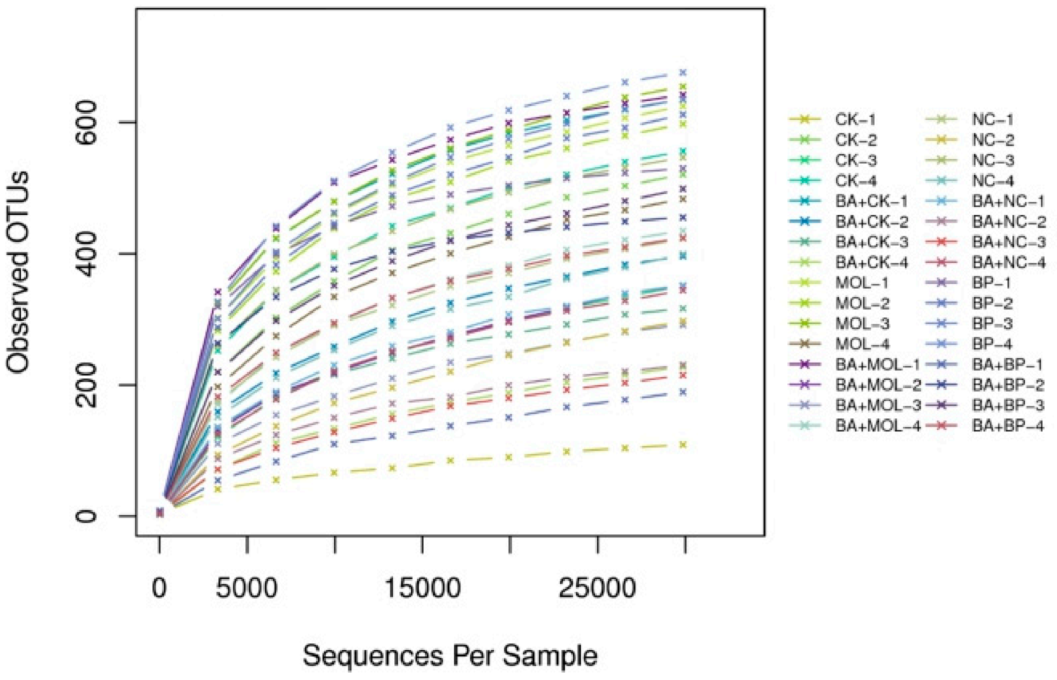

Fig. S1. Rarefaction curves constructed for bacterial 16S rRNA sequences from intestine of tilapia fed with different diets (groups). CK, control diet; BA+CK, *Bacillus amyloliquefaciens* (BA) + control diet; MOL, *Moringa oleifera* diet; BA+MOL, BA + *M. oleifera* diet; NC, *Neolamarckia cadamba* diet; BA + NC, BA+ *N. cadamba* diet; BP, *Broussonetia papyrifera* diet; BA + BP, BA + *B. papyrifera* diet.

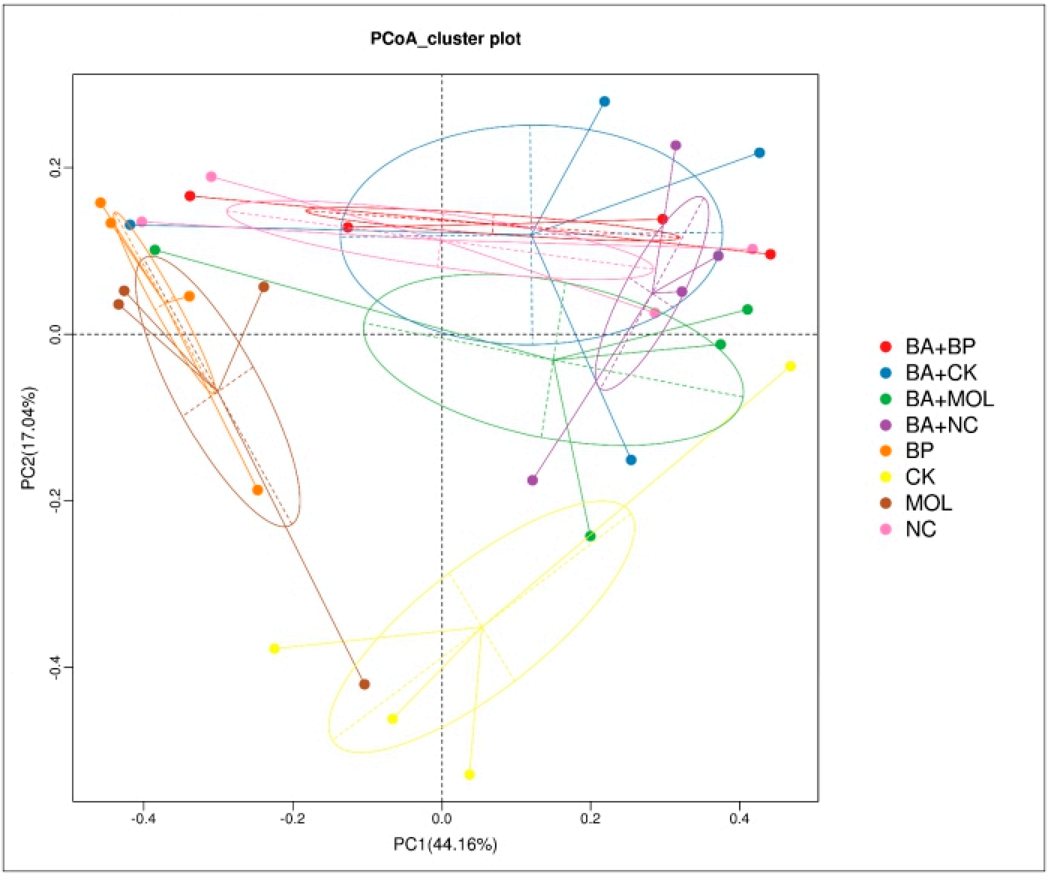

Fig. S2. Beta-diversity measures of the tilapia hindgut bacterial community by principal coordinate analysis based on Brary-Curtis distance matrix. CK, control diet; BA+CK, *Bacillus amyloliquefaciens* (BA) + control diet; MOL, *Moringa oleifera* diet; BA+MOL, BA + *M. oleifera* diet; NC, *Neolamarckia cadamba* diet; BA + NC, BA+ *N. cadamba* diet; BP, *Broussonetia papyrifera* diet; BA + BP, BA + *B. papyrifera* diet.

Table S1 Feed formulation and proximate composition of the eight test feeds (%).

	CK	CK+BA.	MOL	MOL+BA	NC	NC+BA	BP	BP+BA
soybean meal	35.00	35.00	30.00	30.00	35.00	35.00	33.00	33.00
rapeseed meal	28.00	28.00	28.00	28.00	28.00	28.00	28.00	28.00
wheat flour	29.00	29.00	24.00	24.00	27.00	27.00	25.00	25.00
Soybean oil	3.00	3.00	3.00	3.00	3.00	3.00	3.00	3.00
Perilla seed oil	0.80	0.80	0.80	0.80	0.80	0.80	0.80	0.80
Calcium dihydrogen phosphate	2.00	2.00	2.00	2.00	2.00	2.00	2.00	2.00
Vitamin and mineral mixture[a]	1.00	1.00	1.00	1.00	1.00	1.00	1.00	1.00
Methionine	0.40	0.40	0.40	0.40	0.40	0.40	0.40	0.40
Betaine	0.60	0.60	0.60	0.60	0.60	0.60	0.60	0.60
Choline chloride	0.20	0.20	0.20	0.20	0.20	0.20	0.20	0.20
Moringa oleifera Lam. meal[b]			10.00	10.00				
Neolamarckia cadamba meal[b]					2.00	2.00		
Broussonetia papyrifera meal[b]							6.00	6.00
Probiotic		+		+		+		+
Analyzed proximate composition (%)								
Moisture	6.00	5.84	5.30	5.33	5.73	5.62	5.28	5.35
Crude protein	32.70	32.59	32.35	32.27	32.56	32.60	32.55	32.56
Ether extract	5.92	5.92	6.01	5.99	5.84	5.84	5.85	5.83
Crude ash	6.78	7.01	7.13	7.23	7.08	7.48	6.66	6.83

[a] Vitamin mixture (mg/kg premix): thiamine, 0.438; riboflavin, 0.632; pyridoxine·HCl, 0.908; D-pantothenic acid, 1.724; nicotinic acid, 4.583; biotin, 0.211; folic acid, 0.549; vitamin B-12, 0.001; inositol, 21.053; menadione sodium bisulfite, 0.889; retinyl acetate, 0.677; cholecalciferol, 0.116; DL-α-tocopherol-acetate, 12.632; mineral mixture (mg/kg premix): CoCl2·6H2O, 0.074; CuSO4·5H2O, 2.5; FeSO4·7H2O, 73.2; NaCl, 40.0; MgSO4·7H2O, 284.0; MnSO4· H2O, 6.50; KI, 0.68; Na2SeO3, 0.10; ZnSO4·7H2O, 131.93; cellulose, 501.09.

[b]Three kinds of plants were picked in the Arboretum of South China Agricultural University (Guangzhou, China).

CK, control diet; BA+CK, *Bacillus amyloliquefaciens* (BA) + control diet; MOL, *Moringa oleifera* diet; BA+MOL, BA + *M. oleifera* diet; NC, *Neolamarckia cadamba* diet; BA + NC, BA+ *N. cadamba* diet; BP, *Broussonetia papyrifera* diet; BA + BP, BA + *B. papyrifera* diet.

Table S2 Primers used for real-time PCR analysis from Nile tilapia

Gene	Forward and Reverse primers (5′–3′)
TNF-α	F: TAGAAGGCAGCGACTCAA
	R: CCTGGCTGTAGACGAAGT
IL-6	F: ACAGAGGAGGCGGAGATG
	R: GCAGTGCTTCGGGATAGAG
IL-10	F: TGGAGGGCTTCCCCGTCAG
	R: CTGTCGGCAGAACCGTGTCC
C3	F：CAGGCAGGAGGATGTATCGG
	R: TGCCAGCGTCAAGTCTTTTCT
β-actin	F: CAGGGAGAAGATGACCCAGA
	R: CAGGGCATAACCCTAGTAGA

Table S3 Richness and diversity indexes relative to each group (OTU cutoff of 0.03)

Sample ID	Read numbers	Coverage	Number of OUTs	Alpha diversity			
				Ace	Chao1	Shannon	Simpson
CK	128548±4982^{ab}	0.9965±0.0005^{ab}	397±104^{bc}	518±110^{abc}	518±113^{abc}	3.44±0.49^{bc}	0.74±0.05^{abc}
BA+CK	102619±8662^{cd}	0.9967±0.0003^{ab}	406±88^{bc}	520±76^{abc}	516±77^{abc}	3.19±1.05^{c}	0.63±0.17^{bc}
MOL	127005±3650^{ab}	0.9958±0.0003^{b}	605±38^{ab}	719±43^{a}	729±45^{a}	5.33±0.44^{ab}	0.90±0.03^{ab}
BA+MOL	91075±7472^{d}	0.9965±0.0003^{ab}	443±76^{abc}	554±52^{abc}	535±56^{abc}	3.60±0.96^{bc}	0.71±0.11^{abc}
NC	131495±5777^{ab}	0.9965±0.0005^{ab}	430±52^{abc}	708±51^{ab}	710±48^{ab}	5.73±0.13^{a}	0.92±0.02^{a}
BA+NC	109611±3465^{bcd}	0.9973±0.0006^{a}	296±38^{c}	499±57^{bc}	510±63^{bc}	3.22±0.55^{c}	0.69±0.06^{abc}
BP	136424±9481^{a}	0.9963±0.0003^{ab}	625±33^{a}	580±36^{abc}	571±40^{abc}	2.76±0.63^{c}	0.56±0.11^{c}
BA+BP	117898±8933^{abc}	0.9968±0.0005^{ab}	402±70^{bc}	415±53^{c}	399±50^{c}	2.61±0.16^{c}	0.65±0.03^{abc}

The value is the mean ± SE (n = 4). Values with the same superscript in the same column were not significantly different (P> 0.05). OTU = operation classification unit.

CK, control diet; BA+CK, *Bacillus amyloliquefaciens* (BA) + control diet; MOL, *Moringa oleifera* diet; BA+MOL, BA + *M. oleifera* diet; NC, *Neolamarckia cadamba* diet; BA + NC, BA+ *N. cadamba* diet; BP, *Broussonetia papyrifera* diet; BA + BP, BA + *B. papyrifera* diet.
